# Serum afamin levels in predicting gestational diabetes mellitus and preeclampsia: A systematic review and meta-analysis

**DOI:** 10.3389/fendo.2023.1157114

**Published:** 2023-03-22

**Authors:** Ying Yuan, Wenyin He, Xuejiao Fan, Junyu Liang, Zhen Cao, Lei Li

**Affiliations:** ^1^ Department of Clinical Laboratory, The Third Affiliated Hospital of Guangzhou Medical University, Guangzhou, China; ^2^ Zhujiang Hospital of Southern Medical University, Guangzhou, China

**Keywords:** afamin, gestational diabetes mellitus, preeclampsia, meta-analysis, meta-regression analysis, first-trimester

## Abstract

**Objectives:**

The association between biomarkers and the risk of gestational diabetes mellitus (GDM) or preeclampsia (PE) has been extensively studied. However, there is still a lack of convenient, specific, and sensitive indicators for early identification of GMD and PE. Therefore, we conducted a meta-analysis of published articles to investigate the value of afamin circulating levels in the early diagnosis of GDM and PE.

**Methods:**

We searched the PubMed, Embase, Cochrane Library, and Web of Science databases for English studies published before November 16, 2022, that examined the association between afamin and GDM or PE. In addition, we searched Clinicaltrials.gov for the relevant completed and ongoing clinical trials. Pooled standard mean differences (SMDs) and weighted mean differences (WMDs) with 95% confidence intervals (CIs) were used to compare the levels of afamin in different groups.

**Results:**

Eleven studies were included in our analysis (N = 3047 participants: 1195 GDM, 1407 non-GDM, 195 PE, and 250 non-PE). Subgroup analysis based on different blood collection periods found that the plasma afamin levels in pregnant women with GDM in the first trimester were significantly higher than those in healthy pregnant women (SMD = 0.481, 95% CI: 0.280-0.682), but the analysis showed the opposite results in the second and late stages (SMD = 0.292, 95% CI: -0.092-0.676). The plasma afamin levels of pregnant women with PE in the first trimester (SMD = 0.808, 95% CI: 0.558-1.059) and second/third trimesters (SMD = 0.904, 95% CI: 0.570-1.239) were significantly higher than those in healthy pregnant women.

**Conclusion:**

The plasma afamin levels in pregnant women with GDM in the first trimester were significantly higher than those in healthy pregnant women, but the analysis showed the opposite results in the second and third trimesters. The plasma afamin levels in pregnant women with PE in the first, second, and third trimesters were significantly higher than those in healthy pregnant women. Additional large-scale prospective studies are desired to verify these findings, and it is recommended that afamin should be included as a routine diagnostic test for women with GDM and PE.

**Systematic review registration:**

https://www.crd.york.ac.uk/PROSPERO/display_record.php?RecordID=339171, identifier CRD42022339171.

## Introduction

Gestational diabetes mellitus (GDM), one of the most common pregnancy complications, is defined as varying degrees of impaired glucose tolerance for the first time during pregnancy (1, 2). The incidence of GDM is increasing as the obesity rate and the number of elderly parturient women rise globally. According to data released by the International Diabetes Federation (IDF) in 2019, about one-sixth of pregnant women are affected by GDM. GDM poses a serious threat to global public health. In China, the incidence of GDM is about 14.8% (3, 4). GDM causes a variety of adverse pregnancy outcomes, including premature birth, macrosomia, neonatal respiratory distress syndrome, neonatal hypoglycemia, and neonatal hyperbilirubinemia. Besides, GDM significantly increases the risk of type 2 diabetes and cardiovascular disease in postpartum women (5–7). In addition to GDM, preeclampsia (PE) is also a common and serious complication during pregnancy, which is closely related to a variety of adverse pregnancy outcomes. PE leads to heart, brain, kidney, and other organ failures in pregnant women, and obstetrical complications such as placental abruption. Furthermore, PE also leads to fetal growth restriction and fetal distress, which seriously endangers the health of pregnant women and fetuses. PE is one of the important causes of maternal death (8), with a global incidence of about 3%-5%, responsible for 10% of global neonatal and perinatal mortality (9, 10).

At present, the screening of GDM generally is conducted in the second and third trimesters of pregnancy (24-28 weeks), and the oral glucose tolerance test (OGTT) is used for auxiliary diagnosis. Research data show that intervention treatment for pregnant women with GDM in the second and third trimesters helps control short-term complications, but does not alleviate long-term complications. Lifestyle interventions for pregnant women before 15 weeks of gestation reduce the risk of GDM by 20% (11–13). The main features and diagnostic basis of PE are new-onset hypertension [systolic blood pressure ≥ 140 mmHg and (or) diastolic blood pressure ≥ 90 mmHg] and proteinuria (≥ 300 mg/24 h) after 20 weeks of pregnancy. The timing of diagnosis and treatment of PE depends on the clinical manifestations caused by target organ damage and laboratory indicators. Delayed identification may miss the best time for an intervention. As a result, finding biomarkers for predicting GDM and PE and effective screening, diagnosis, and treatment for pregnant women can help reduce the occurrence of maternal and fetal complications.

Afamin is a polysaccharide-protein that is mainly secreted by the liver and widely exists in human plasma. Elevated plasma afamin levels are closely associated with oxidative stress, insulin resistance, and metabolic syndrome (14). There have been a small number of studies on the predictive effect of plasma afamin levels during pregnancy on GDM and PE. Considering that the sample size of the existing studies is small, and the source and sampling time of the samples are not completely consistent, we conducted a meta-analysis to determine whether there is a difference in the plasma afamin levels between pregnant women with GDM or PE and normal pregnant women, to provide a medical basis for the role of afamin in the prediction of GDM and PE.

## Methods

### Search strategy and selection criteria

This systematic review and meta-analysis are reported in accordance with the Preferred Reporting Items for Systematic Reviews and Meta-Analyses (PRISMA) Statement and was registered on the International Prospective Register of Systematic Reviews (number CRD42022339171).

We selected relevant English studies published before November 16, 2022, in PubMed, Embase, Cochrane Library, and Web of Science databases. Bot free words and MeSH terms were used, such as “afamin” and (“Diabetes, Gestational” or “Pre-Eclampsia”). The complete search used for PubMed was presented in the **Supplementary File**. The literature was independently searched by two researchers, and any discrepancies were solved by a discussion with a third researcher. In addition, we searched Clinicaltrials.gov (https://clinicaltrials.gov) for the relevant completed and ongoing clinical trials, and references in other related articles were also scanned to find eligible studies.

### Study selection and data extraction

#### Inclusion criteria

Studies (1) included normal pregnant women without pregnancy complications and pregnant women with gestational diabetes or preeclampsia; (2) evaluated the relationship between plasma afamin levels and gestational diabetes or preeclampsia (correlation); (3) provided analytical data on plasma afamin level outcome; (4) English literature; (5) the type of study was not limited (RCTs, cohort studies, case-control studies, cross-sectional studies, etc.).

#### Exclusion criteria

Studies (1) did not evaluate the relationship between gestational diabetes or preeclampsia and afamin as the main factor of concern; (2) included cases of gestational diabetes combined with previous type 1 diabetes or type 2 diabetes; (3) included insufficient data provided for statistical analysis; (4) combined afamin with other biomarkers for diagnosis; (5) were case reports, reviews, meeting abstracts and letters to the editor, etc.; (6) included non-human study objectives; (7) were not in English or repeatedly published.

Two independent investigators (YY, LL) reviewed study titles and abstracts, and studies that satisfied the inclusion criteria were retrieved for full-text assessment. Trials selected for detailed analysis and data extraction were analyzed by two investigators (YY and LL); disagreements were resolved by a third investigator (CZ).

We extracted the following data from the eligible studies: author, published year, country, the number of participants (cases/controls), age, time of afamin measurement, assay method, afamin levels (mean [SD]), diagnostic criteria for GDM/PE and Newcastle and Ottawa scale (NOS) scores. Two independent reviewers (YY, LL) assessed the risk of bias in the included studies according to the PRISMA recommendations.

### Statistical analysis

We analyzed afamin levels as continuous variables for statistical analysis and reported them as the mean and standard deviation (SD). Some studies provide medians and interquartile ranges, which we estimated as means and SDs. Afamin levels in healthy pregnant women and women with GDM or PE were compared by weighted mean differences (WMDs) or standardized mean differences (SMDs) with 95% confidence intervals (95% CIs). The meta-analyses and meta-regression analyses were conducted using Stata software version 15.0. Spearman’s correlation coefficient was used for heterogeneity analysis to evaluate the threshold effect, and Cochran’s Q, *I^2^* test was used to evaluate the on-threshold effect. When *p* < 0.05 or *I^2^* > 50%, the heterogeneity was considered significant, and a random-effects model was used to pool the statistics; otherwise, a fixed-effects model was used. Sensitivity tests and meta-regression were used to analyze the causes of heterogeneity, and the sensitivity analyses were performed by omitting one study at a time to compute the pooled effect size of the remaining studies to evaluate whether the results were markedly affected by a single study. Forest plots and funnel plots were used for assessing the overall effect size and evaluating publication bias, respectively. Possible publication biases were evaluated using funnel plots. We assessed funnel plot asymmetry using Begg’s and Egger’s tests, and defined significant publication bias as a *p-value* < 0.05.

## Results

**Figure 1:**
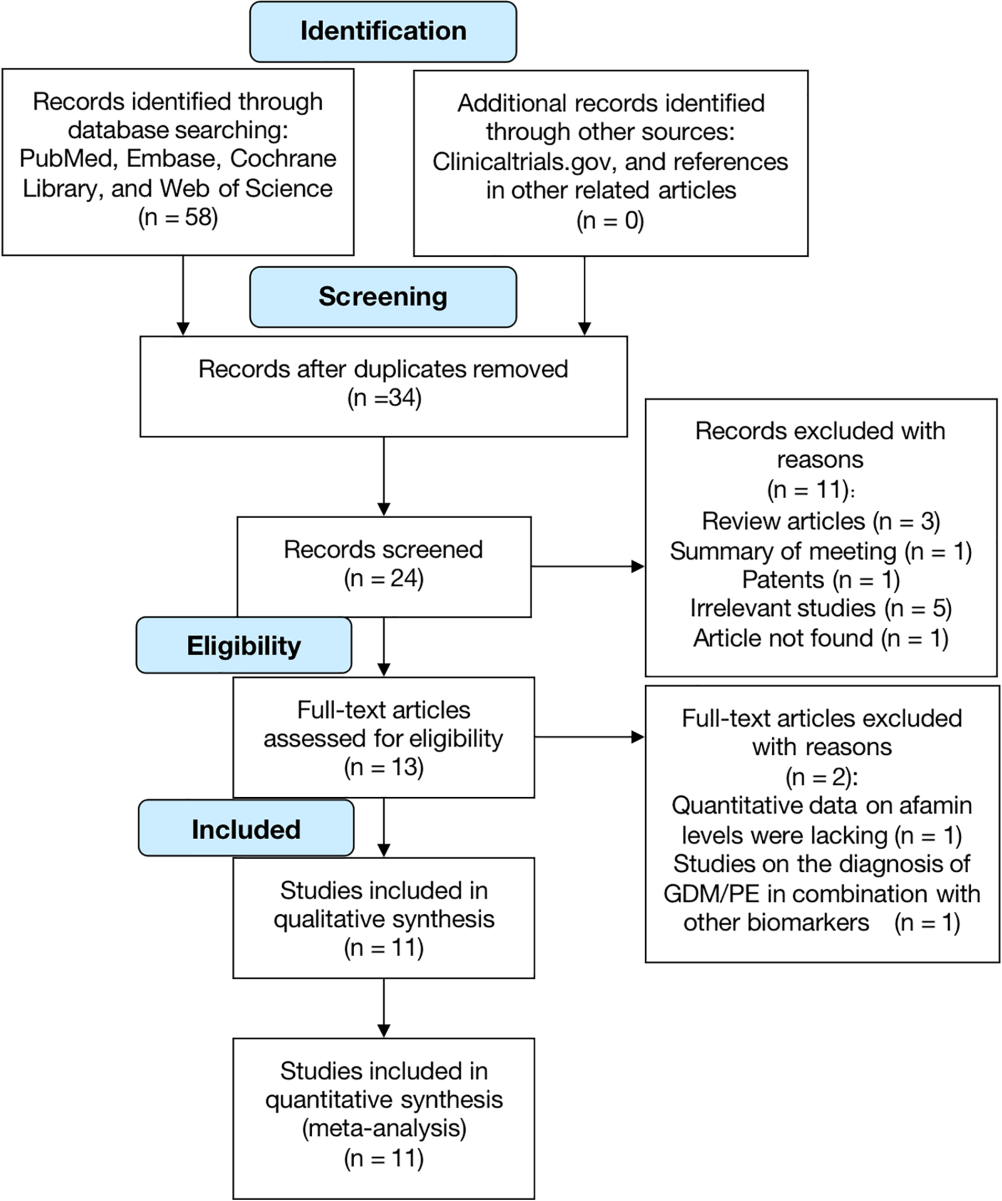
Flow chart of literature selection.

The initial search identified 58 studies, and no additional records were identified through other sources. After excluding duplicate studies, irrelevant titles, and abstracts, 13 studies were obtained, 11 of which were included in our analysis (**Table 1**). **Figure 1** summarizes the process of literature search and screening, and the **Supplementary File** presents the complete search formula of the PubMed database. The 11 eligible studies were all published between 2014 and 2022 (6 of which were published between 2021 and 2022), involving a total of 3047 pregnant women (1195 pregnant women with GDM, 195 pregnant women with PE, and 1657 healthy pregnant women). The 11 included studies were from 5 countries: 3 from Austria, 3 from Turkey, 2 from Germany, 2 from China, and 1 from Iraq. Of the 11 studies, 4 focus on PE, 5 on GDM, and 2 on both PE and GDM. The timing of blood collection included the first trimester (6 studies), first and second trimesters (1 study), second trimester (2 studies), and third trimester (2 studies).

**Table 1:** Characteristics of available studies relating afamin levels to GDM or PE risk.

Number	Author	Year	Country	Participants (cases/controls)	Age(cases/controls)	Disease	Diagnostic criteria	Time of afamin measurement	Assay method	NOS scores
1	Michael Hubalek	2014	Austria	13/13	NA	PE	ISSHP	First-trimester	ELISA	8
2	Angela Köninger	2018	Germany	59/51	34.36 ± 5.26/32.65 ± 4.79	GDM	DGGG	First-trimester	ELISA	7
3	Angela Köninger	2018	Germany	39/98	33.15 ± 4.97/32.79 ± 4.81	PE	ISSHP	First-trimester	ELISA	7
4	Allessandra Tramontana, Benjamin Dieplinger	2018	Austria	209/209	32(28-36)/31(29-35)	GDM	IADPSG	First-trimester	ELISA	8
				30/30	32(23-36)/32(29-35)	PE	ISSHP	First-trimester	ELISA	
5	Allessandra Tramontana, Eleonore Pablik	2018	Austria	170/170	31.33 ± 5.40/NA	GDM	IADPSG	First-trimester	ELISA	8
				33/33	29.58 ± 6.77/NA	PE	ISSHP	First-trimester	ELISA	
6	Nil Atakul	2021	Turkey	49/40	32.04 ± 5.45/27.23 ± 5.30	GDM	IADPSG	Third-trimester	ELISA	7
7	Hasan Eroğlu	2021	Turkey	43/44	26.93 ± 6.08/25.98 ± 4.07	GDM	ACOG	First-trimester	ELISA	7
8	Xuechun Wang	2021	China	607/833	30.39 ± 3.66/28.42 ± 3.30	GDM	ADA	First-trimester	ELISA	7
9	Canan Soyer Çalışkan	2021	Turkey	39/46	28.97 ± 6.00/29.07 ± 5.97	PE	ISSHP	Second-trimester	ELISA	7
10	Anas Hashim Sadek	2021	Iraq	41/30	29.05 ± 6.21/29.70 ± 7.50	PE	ISSHP	Third-trimester	ELISA	7
11	Qian Li	2022	China	58/60	29.57 ± 2.94/29.50 ± 2.27	GDM	IADPSG	Second-trimester	ELISA	8

NA, not applicable; ELISA, enzyme- linked immunosorbent assay; NOS, Newcastle Ottawa Scale; ISSHP, International Society for the Study of Hypertension in Pregnancy; DGGG, German Society of Gynecology and Obstetrics; IADPSG, International Association of Diabetes and Pregnancy Study Group; ACOG, American College of Obstetriciansand Gynecologists; ADA, Americn Diabetos Association.

**Figure 2:**
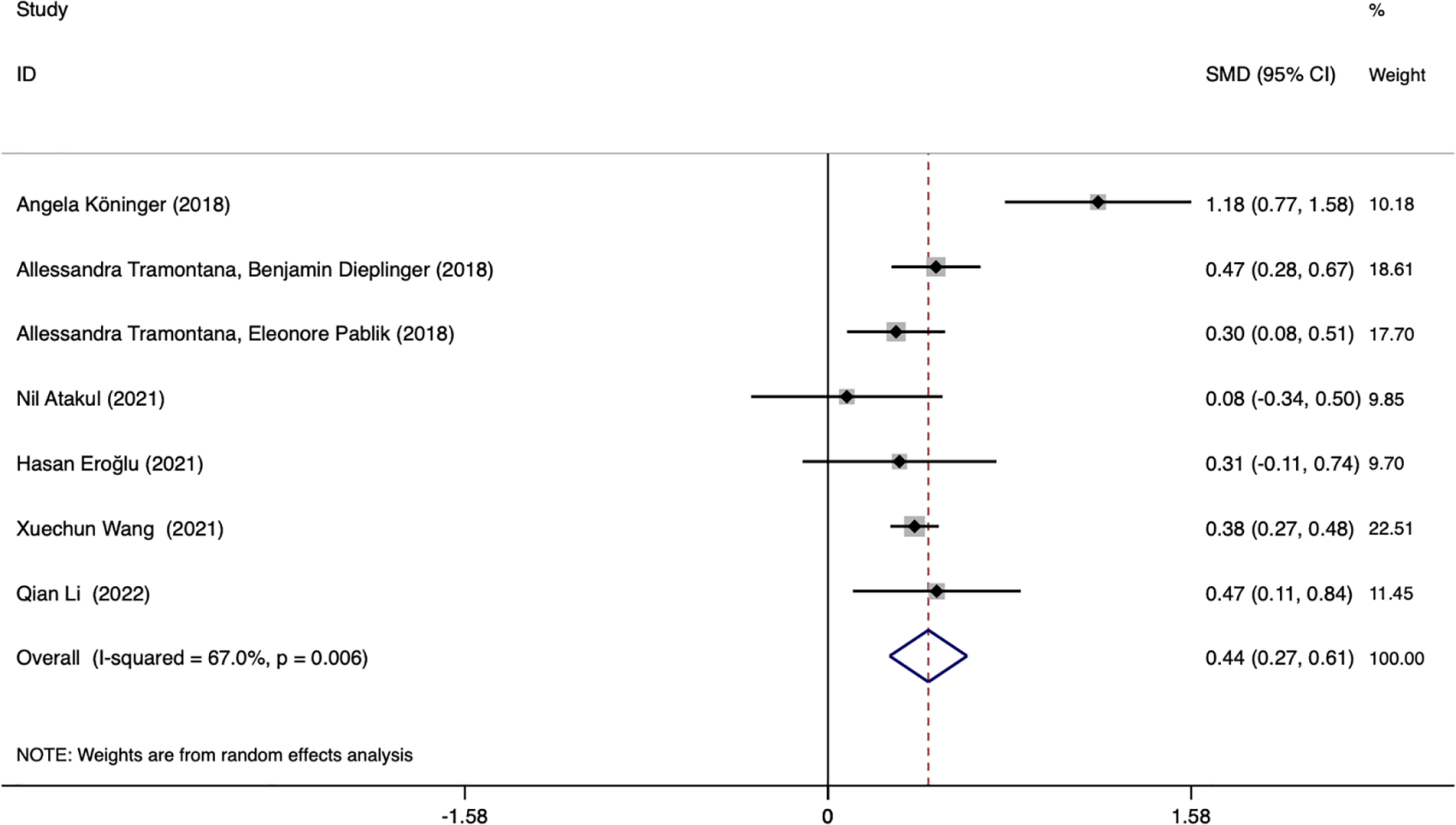
Forest plot of meta-analysis of the predictive value of serum afamin levels for gestational diabetes mellitus. SMD, standard mean difference; CI, confidence intervals.

**Figure 3:**
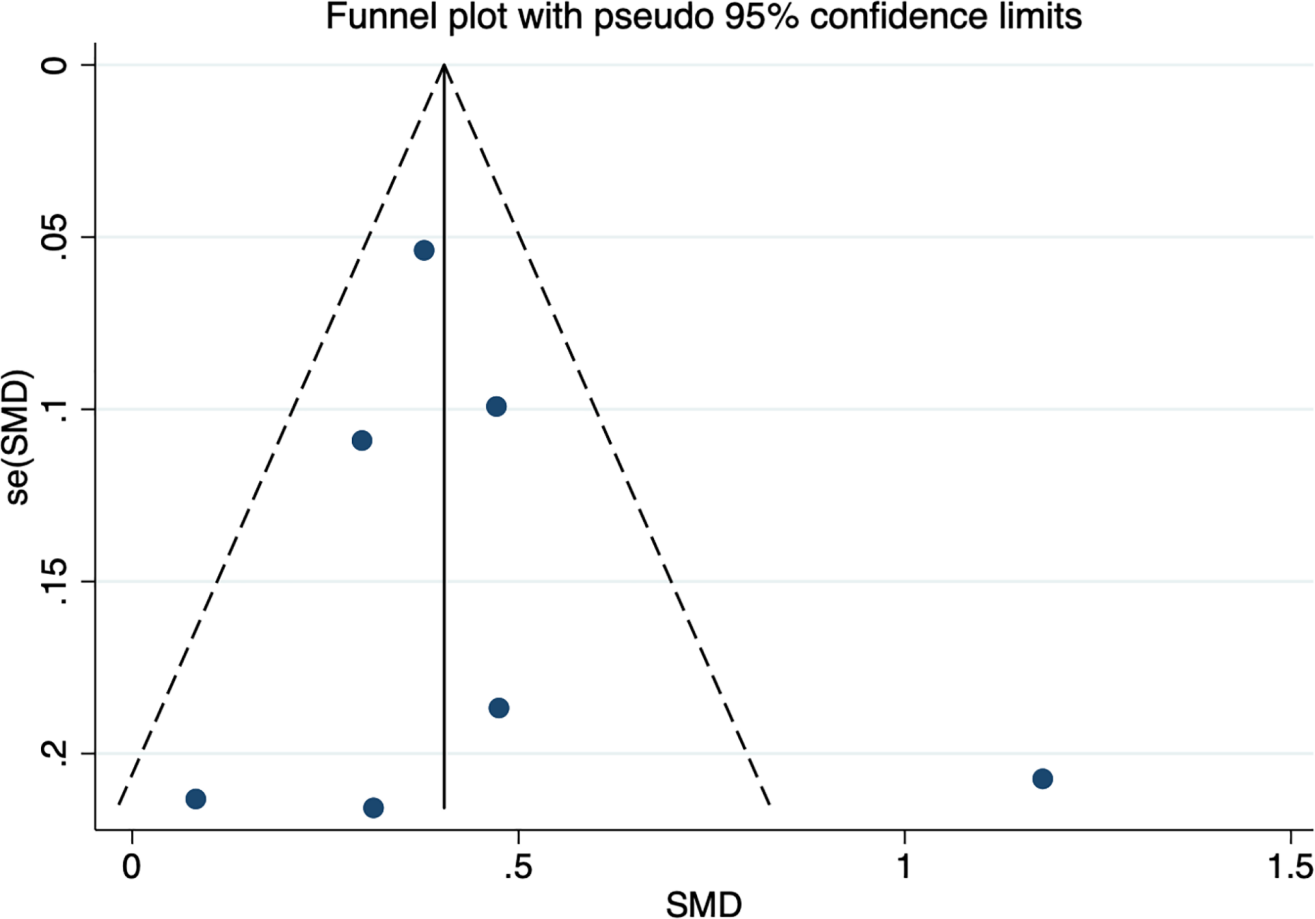
Publication bias funnel plot for the meta-analysis of the standard mean difference for serum afamin levels and gestational diabetes mellitus. SMD, standard mean difference; se(SMD), standard error of standard mean difference.

Seven studies assessed whether the afamin levels differed between GDM and healthy pregnant women (2602 total: 1407 healthy pregnant women, 1195 GDM pregnant women). There was significant heterogeneity among the included studies. (*I^2^* = 67.0%, *p* = 0.006) (**Figure 2**). The random effect model was used for meta-analysis, and the results showed that the serum afamin levels of pregnant women with GDM were higher than those of healthy pregnant women (SMD = 0.438, 95% CI: 0.269-0.607), and the difference was statistically significant *p* = 0.000. The publication bias was tested by Egger, and the funnel plot showed a roughly symmetrical shape. The results suggested that no significant publication bias was found in the studies included in the meta-analysis (*t* = 0.58, *p* = 0.589). The results were shown in **Figure 3**.

Subgroup analysis was performed based on the ethnicity of the included population, GDM diagnostic criteria, and blood collection period to find the source of heterogeneity. Subgroup analysis showed that the source of heterogeneity may be the studies reporting the period of blood collection in the first trimester (*I^2^* = 74.6%). Further analysis of these studies found that the Angela Köninger (2018, Germany) literature was the source of heterogeneity. Gynecologist Angela Köninger diagnosed the GDM case according to current German obstetric care guidelines. After the study was excluded, the heterogeneity was significantly reduced (*I^2^* = 0.0%), and the results were the same and stable SMD = 0.374, 95% CI: 0.294-0.454, *p* = 0.000.

**Figure 4:**
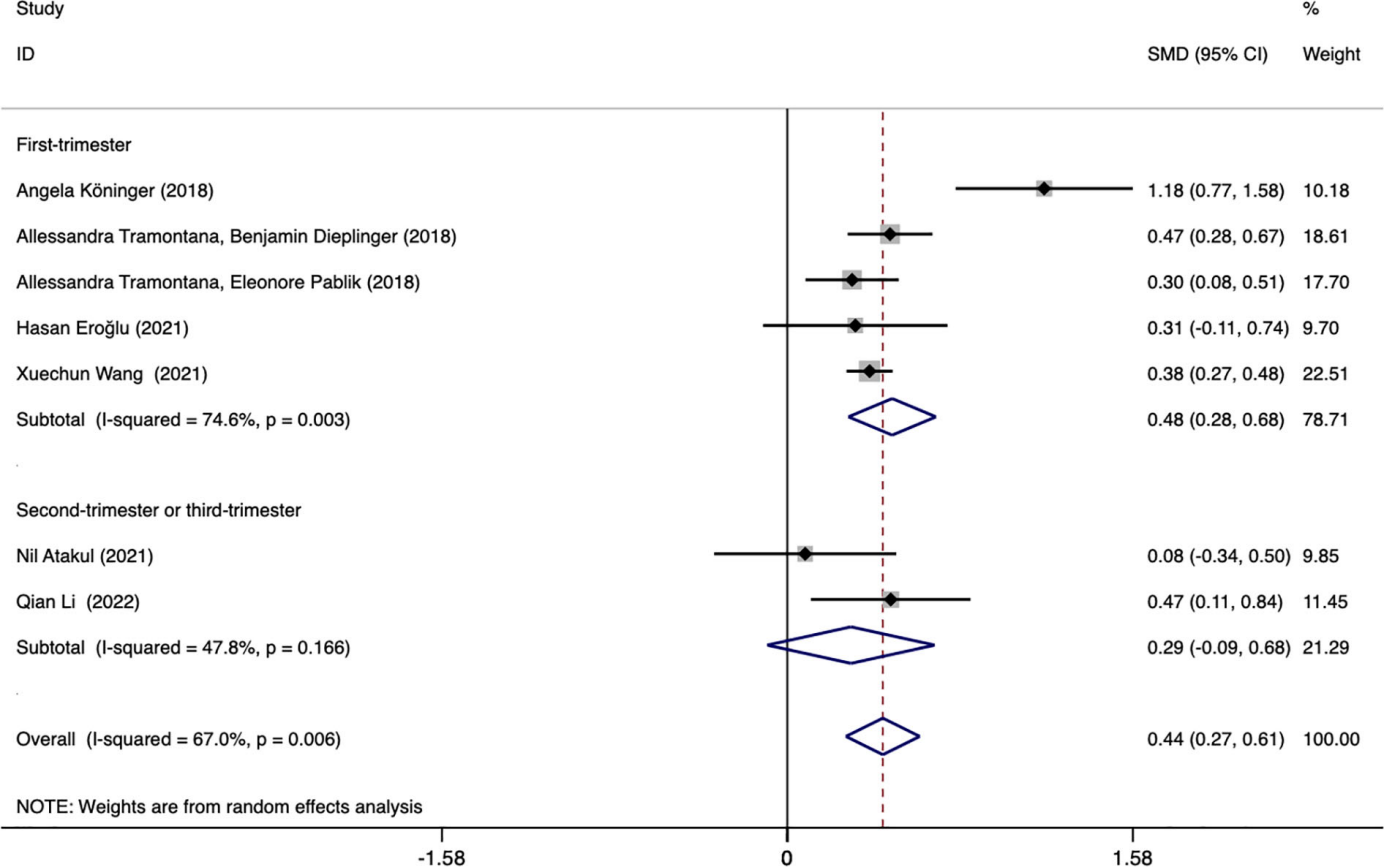
Forest plot was used to evaluate the value of serum afamin levels in predicting gestational diabetes mellitus in the first trimester group and the second/third trimester group. SMD, standard mean difference; CI, confidence intervals.

Subgroup analysis based on different blood collection periods found that the source of heterogeneity may be the study reporting the first-trimester blood collection period, the results of this subgroup showed that the plasma afamin levels of pregnant women with GDM in the first trimester were significantly higher than those of healthy pregnant women (SMD = 0.481, 95% CI: 0.280-0.682); the results of another subgroup showed that there was no significant difference in serum afamin levels between pregnant women with GDM in the second and late stages of pregnancy and healthy pregnant women (SMD = 0.292, 95% CI: -0.092-0.676), as shown in **Figure 4**.

Multivariate regression analysis was performed in three GDM-related studies [Tramontana et al., (15); Tramontana, et al., (16); Wang et al., (17)]. The results of the meta-regression analysis showed that there was no significant difference in serum afamin levels between pregnant women with GDM and healthy pregnant women (*p* = 0.310, ES = 1.009, 95% CI: 0.992-1.026). The heterogeneity of the three studies was significant (*I^2^* = 86.8%) and the publication bias was not significant (*t* = 7.45, *p* = 0.085).

**Figure 5:**
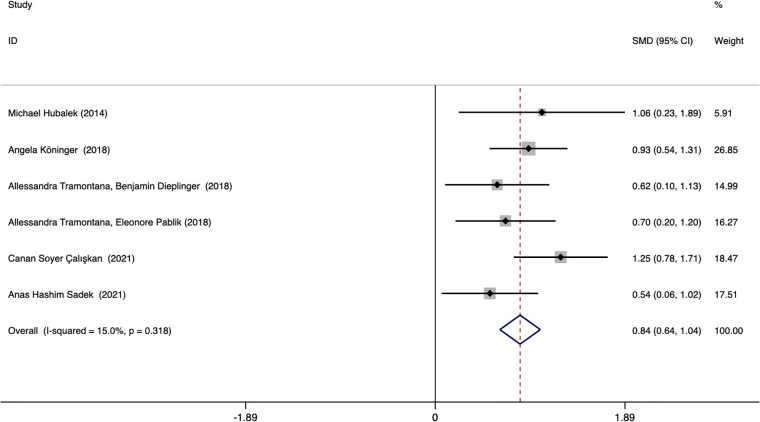
Forest plot of meta-analysis of the predictive value of serum afamin levels for preeclampsia. SMD, standard mean difference; CI, confidence intervals.

**Figure 6:**
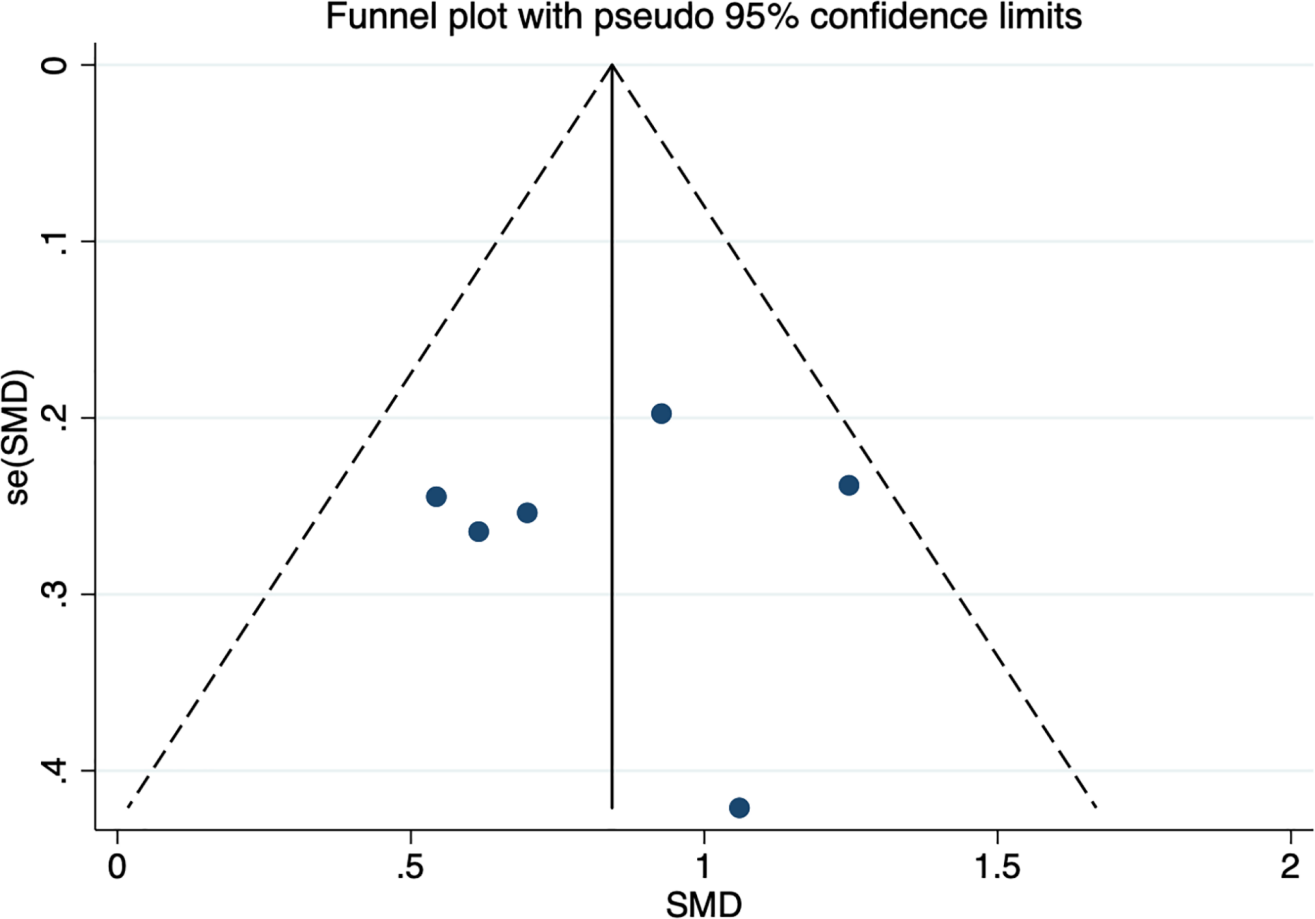
Publication bias funnel plot for the meta-analysis of the standard mean difference for serum afamin levels and preeclampsia. SMD, standard mean difference; se(SMD), standard error of standard mean difference.

Six studies assessed whether the afamin levels differed between PE and healthy pregnant women (445 total: 250 healthy pregnant women, 195 PE pregnant women). There was no significant heterogeneity across the included studies (*I^2^* = 15.0%, *p* = 0.000) (**Figure 5**). The fixed effect model was used for meta-analysis, and the results showed that the serum afamin levels of pregnant women with PE were higher than those of healthy pregnant women (SMD = 0.843, 95% CI: 0.642-1.044), and the difference was statistically significant *p* = 0.000. The publication bias was tested by Egger, and the funnel plot showed a roughly symmetrical shape. The results suggested that no significant publication bias was found in the studies included in the meta-analysis (*t* = -0.03, *p* = 0.979). The results were shown in **Figure 6**.

**Figure 7:**
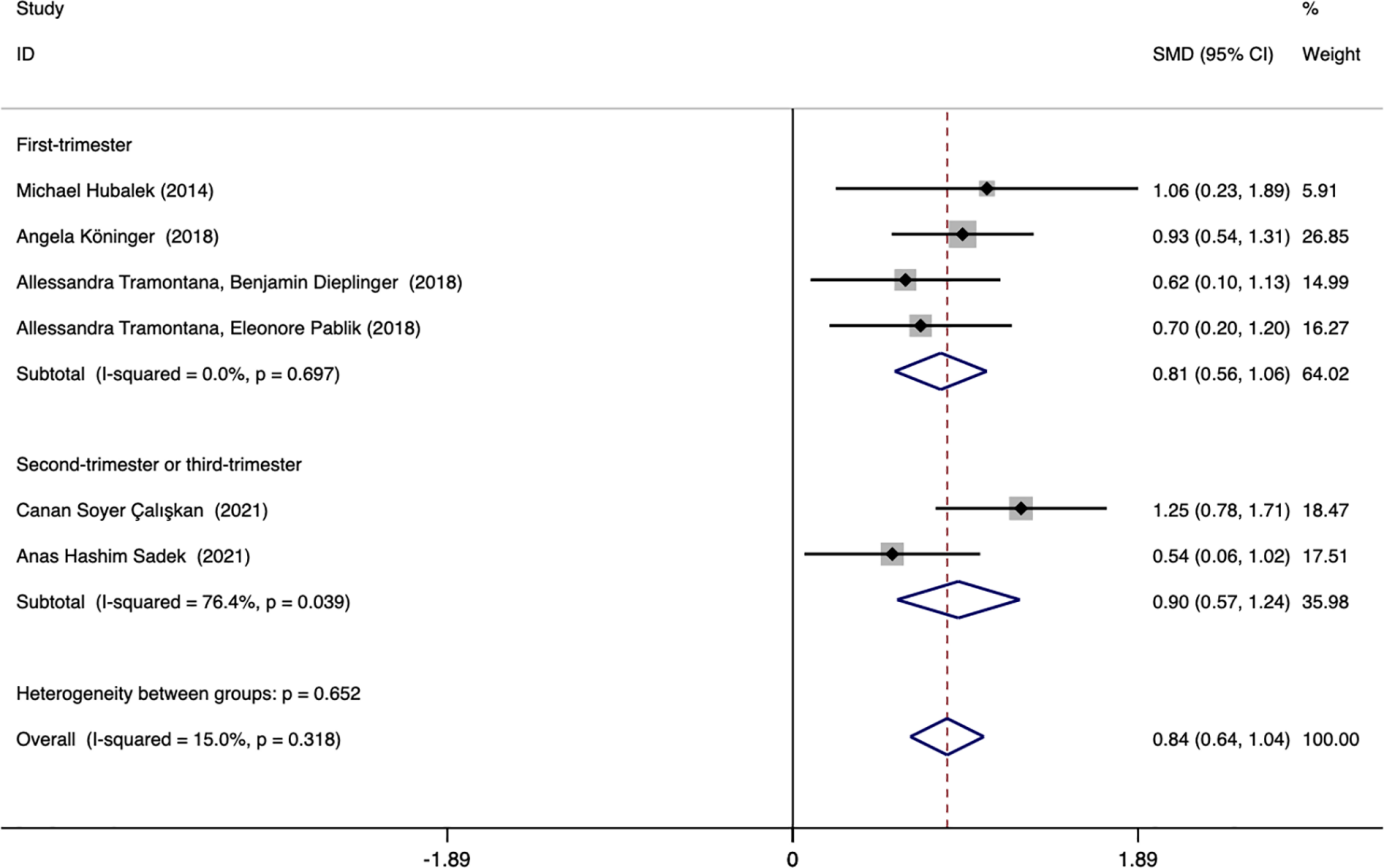
Forest plot was used to evaluate the value of serum afamin levels in predicting preeclampsia in the first trimester group and the second/third trimester group. SMD, standard mean difference; CI, confidence intervals.

Subgroup analysis was performed according to the ethnicity and blood collection period of the included population to explore the source of heterogeneity. The results showed that the heterogeneity may come from studies reporting the time of blood collection in the second and third trimesters (*I^2^* = 76.4%). Further analysis found that Canan Soyer Çalışkan (2021, Turkey) was the source of heterogeneity. After excluding this literature, the heterogeneity was significantly reduced (*I^2^* = 0.0%), and the result remained unchanged. Subgroup analysis based on the time of blood collection found that the plasma afamin levels of pregnant women with PE in the first trimester were significantly higher than those in healthy pregnant women (SMD = 0.808, 95% CI: 0.558-1.059); the plasma afamin levels in pregnant women with PE in the second and third trimesters were significantly higher than those in healthy pregnant women (SMD = 0.904, 95% CI: 0.570-1.239) (**Figure 7**).

Multivariate regression analysis was performed in 4 PE-related studies [Köningeret al., (18); Tramontana et al., (15); Tramontana et al., (16); Çalışkan et al., (19)]. The meta-regression analysis showed that there was a significant difference in serum afamin levels between pregnant women with PE and healthy pregnant women (*p* = 0.015, ES = 3.089, 95% CI: 1.244-7.666) The heterogeneity of the 4 studies was significant (*I^2^* = 90.6%), and there was no significant publication bias (t = 3.80, *p* = 0.063).

## Discussion

GDM and PE are increasingly serious global public health problems, which can cause a variety of adverse pregnancy outcomes and affect the health of mothers and infants. Related studies have shown that timely intervention and treatment of GDM and PE can effectively improve pregnancy outcomes (20). At present, the screening and diagnosis of GDM and PE are mainly carried out in the second and third trimesters of pregnancy, and the commonly used GDM diagnostic method (OGTT test) requires a series of standardized procedures such as fasting, which is cumbersome. Therefore, it is of great significance to find biomarkers of GDM and PE for convenient and economical early screening and diagnosis. This topic has attracted extensive attention from scholars around the world.

Afamin is a polysaccharide-protein mainly secreted by the liver, which can play an important physiological role as a vitamin E carrier in plasma and other body fluids (21). Studies have shown that elevated plasma afamin levels are closely related to obesity, hyperglycemia, insulin resistance, and metabolic syndrome (22–25). However, little is known about the pathophysiology of afamin. The role of afamin in hypertensive disorder complicated pregnancy (HDCP) and GDM is rarely explored, while most studies focus on the predictive effect of serum afamin levels in early pregnancy on HDCP and GDM. Tramontana et al. (15) found that the plasma afamin levels of PE patients in the first trimester of pregnancy were significantly higher than those of healthy pregnant women, and elevated plasma afamin levels in the first trimester of pregnancy (> 65 mg/L) was a strong and independent risk factor for the development of preeclampsia in late pregnancy. (RR = 24.58, 95% CI: 2.82-214.12, *p* = 0.004). Köninger et al. (26) found that the plasma afamin levels of GDM patients in the first trimester of pregnancy were significantly higher than those of healthy pregnant women, suggesting that afamin may be a new marker for predicting GDM. However, whether elevated plasma afamin levels in early pregnancy can be used as an independent predictor of GDM is not yet clear, and the specific cut-off value of afamin levels still needs further exploration. Based on the above research basis, we conducted a meta-analysis of published articles to investigate the value of plasma afamin levels in the early diagnosis of GDM and PE.

In the process of exploring the correlation between afamin and the risk of GDM, we included 7 studies and performed subgroup analysis according to the detection period of afamin, but the conclusions among the subgroups were inconsistent. As most of the included studies focused on the first trimester, and only two studies reported the second and third trimester data, more studies on the first, second and third trimesters are required to further analyze and investigate whether the significantly increased afamin levels are valuable for the prediction of GDM only in the first trimester. Egger’s test showed no significant publication bias (*t* = 0.58, *p* = 0.589), and the funnel plot was roughly symmetrical, except for the article by A. Koninger et al. (26), published in 2018 (No. 2 in **Table 1**). It was speculated that the reason may be the lack of unified diagnostic criteria for GDM. Among the seven articles included in the analysis, the diagnostic criteria for pregnant women with GDM in this article were derived from the German Society of Gynecology and Obstetrics, with four studies based on the criteria provided by the International Association of Diabetes and Pregnancy Study Group, one based on the criteria provided by the American College of Obstetriciansand Gynecologists, and one based on the criteria provided by the Americn Diabetos Association. Three studies (first trimester) were related to GDM and reported multivariate regression analysis. We performed a meta-regression analysis on these three studies, and the results showed that there was no significant difference in serum afamin levels between pregnant women with GDM and healthy pregnant women (*p* = 0.310, ES = 1.009, 95% CI: 0.992-1.026). After adjusting potential factors such as maternal age, gestational age, fetal birth weight, and maternal plasma triglyceride and HCG levels, there was no significant difference in serum afamin levels between pregnant women with GDM and healthy pregnant women, which was inconsistent with the conclusions obtained from the multivariate regression analysis of a single study. The data of our study do not support that the elevated serum afamin levels in the first trimester are independent predictors of GDM, but this conclusion needs to be confirmed or updated by a large number of further research.

Six studies explored the association between afamin and the risk of PE, and we performed a subgroup analysis of these 6 studies according to the detection period of afamin, the conclusions obtained among the subgroups were consistent. Four studies (first trimester: 3, second trimester: 1) were related to PE, and reported multivariate regression analysis. Meta-regression analysis was performed on 4 studies, and the results showed that there was a significant difference in serum afamin levels between pregnant women with PE and healthy pregnant women (*p* = 0.015, ES = 3.089, 95% CI: 1.244-7.666). After adjusting potential factors such as BMI, maternal age, gestational age, fetal birth weight, and maternal plasma HCG and PAPP-A levels, there were still significant differences in serum afamin levels between PE pregnant women and healthy pregnant women. Therefore, it can be concluded that elevated serum afamin levels during the first and second trimesters are independent predictors of PE, which is consistent with the findings reported by Tramontana et al. (15)

As of now, there is only 1 published meta-analysis study related to the topic of this study. Zixin Cai et al. (27) explored the correlation between liver factor levels and the risk of gestational diabetes. The results showed that the plasma afamin levels of pregnant women with GDM were significantly higher than those of healthy pregnant women (SMD = 0.58, 95% CI: 0.24-0.93), which was consistent with the conclusion of this study. A total of 5 afamin-related studies were included in this study, of which 4 studies had blood collection in the first trimester, and 1 study had population blood collection before pregnancy. The subgroup analysis was carried out according to the period of blood collection, and the conclusion of the subgroup in the first trimester was consistent with the results of this study. Based on this literature, our study added 3 research data published in 2021 and 1 research published in 2022 into the analysis, which further expanded the sample size, supplemented the data in recent years, and laid the foundation for follow-up research. On the other hand, most of the data included in this study were obtained in the first trimester. In the future, studies focusing on different trimesters can be included for subgroup analysis to explore the plasma afamin levels of pregnant women with GDM or PE in the first, second, and third trimesters, and even before pregnancy, to explore whether there is a difference in afamin levels between healthy pregnant women and pregnant women with GDM in the same period, and to further supplement and improve the previous conclusions through meta-regression analysis.

In addition to afamin, previous studies have suggested that some adipocyte-derived markers, placenta-derived markers, and inflammatory markers also have the potential to predict GDM or PE to a certain extent, but their results are not completely consistent (28–31). Y. Uchida et al. (32) found that a 3-protein combination biomarker (afamin, fibronectin, and sex hormone-binding globulin) could effectively predict during gestational weeks 14-24 whether pregnant women would subsequently develop PE, and its predictive performance was favorable. X. Wang et al. (17) concluded that the prediction accuracy of age combined with afamin, triglycerides, and platelet/lymphocyte ratio was higher than any single factor. Hence, it can be inferred that combining multiple biomarkers may achieve higher specificity and sensitivity. Additional in-depth prospective and multi-center studies are needed to explore whether afamin can be combined with other biomarkers to predict GDM or PE at an earlier stage.

In conclusion, our study showed that, the plasma afamin levels of pregnant women with GDM were significantly higher than those of healthy pregnant women in the first trimester; and compared with healthy pregnant women without pregnancy complications, pregnant women with PE in the same period had higher plasma afamin levels. Detection of afamin in early pregnancy can predict the risk of GDM and PE in middle and late pregnancy to a certain extent, to take timely intervention and treatment measures.

The limitations of this study are as follows: the total number of existing relevant studies is relatively small, the sample size is small, the number of studies included in meta-regression analysis and subgroup analysis is small, and the representativeness of the conclusions needs to be further verified by more relevant studies in the future. On the other hand, because PE can develop rapidly and worsen, there is still a lack of biomarkers at this stage to closely monitor and accurately judge the condition of PE patients. In addition, blood glucose monitoring is not good enough to achieve the purpose of preventing and treating GDM and its complications, as well as monitoring the efficacy (33, 34). At present, relevant studies focus on predicting risk value, and there are still few studies reporting the impact of afamin on the severity of PE and GDM, monitoring effect, and maternal and fetal pregnancy outcomes. In the future, additional research is required to further explore whether afamin has the potential to provide greater help for the diagnosis of early PE and GDM.

## Data availability statement

The original contributions presented in the study are included in the article/**Supplementary Material**. Further inquiries can be directed to the corresponding author.

## Author contributions

Conceptualization: LL and YY; Methodology: LL and YY; Formal analysis and investigation: LL, YY, and WH; Writing original draft preparation: YY and WH; Writing review and editing: YY, XF, JL, and ZC; Funding acquisition: LL; Resources: LL; Supervision: LL. and all authors commented on previous versions of the manuscript. All authors contributed to the article and approved the submitted version.
