# Supplementary material for: Serum afamin levels in predicting gestational diabetes mellitus and preeclampsia: A systematic review and meta-analysis
Source: Front Endocrinol (Lausanne). 2023 Mar 22;14:1157114. doi: 10.3389/fendo.2023.1157114 (PMC10073667; doi:10.3389/fendo.2023.1157114)
Supplement: Supplementary file 1 [file Table_1.docx]

PubMed search formula

#1) “Search ("Diabetes, Gestational"[Mesh]) OR (((((((Diabetes, Gestational[Title/Abstract]) OR (Diabetes, Pregnancy-Induced[Title/Abstract])) OR (Diabetes, Pregnancy Induced[Title/Abstract])) OR (Pregnancy-Induced Diabetes[Title/Abstract])) OR (Gestational Diabetes[Title/Abstract])) OR (Diabetes

Mellitus, Gestational[Title/Abstract])) OR (Gestational Diabetes

Mellitus[Title/Abstract]))

#2) “Search ("Pre-Eclampsia"[Mesh]) OR (((((((((((((((((((((((((((((((((((Pre- Eclampsia[Title/Abstract]) OR (Pre Eclampsia[Title/Abstract])) OR (Preeclampsia[Title/Abstract])) OR (Pregnancy Toxemias[Title/Abstract])) OR (Pregnancy Toxemia[Title/Abstract])) OR (Toxemia, Pregnancy[Title/Abstract])) OR (Edema-Proteinuria-Hypertension Gestosis[Title/Abstract])) OR (Edema Proteinuria Hypertension Gestosis[Title/Abstract])) OR (Gestosis, Edema-Proteinuria- Hypertension[Title/Abstract])) OR (Hypertension-Edema-Proteinuria

Gestosis[Title/Abstract])) OR (Gestosis, Hypertension-Edema-

Proteinuria[Title/Abstract])) OR (Hypertension Edema Proteinuria

Gestosis[Title/Abstract])) OR (Toxemia Of Pregnancy[Title/Abstract])) OR (Of Pregnancies, Toxemia[Title/Abstract])) OR (Of Pregnancy, Toxemia[Title/Abstract])) OR (Pregnancies, Toxemia Of[Title/Abstract])) OR (Pregnancy, Toxemia

Of[Title/Abstract])) OR (Toxemia Of Pregnancies[Title/Abstract])) OR (EPH Complex[Title/Abstract])) OR (EPH Toxemias[Title/Abstract])) OR (EPH Toxemia[Title/Abstract])) OR (Toxemia, EPH[Title/Abstract])) OR (Toxemias, EPH[Title/Abstract])) OR (EPH Gestosis[Title/Abstract])) OR (Gestosis,

EPH[Title/Abstract])) OR (Toxemias, Pregnancy[Title/Abstract])) OR (Preeclampsia Eclampsia 1[Title/Abstract])) OR (1, Preeclampsia Eclampsia[Title/Abstract])) OR (1s, Preeclampsia Eclampsia[Title/Abstract])) OR (Eclampsia 1,

Preeclampsia[Title/Abstract])) OR (Eclampsia 1s, Preeclampsia[Title/Abstract])) OR (Preeclampsia Eclampsia 1s[Title/Abstract])) OR (Proteinuria-Edema-Hypertension

Gestosis[Title/Abstract])) OR (Gestosis, Proteinuria-Edema-

Hypertension[Title/Abstract])) OR (Proteinuria Edema Hypertension

Gestosis[Title/Abstract]))

#3) “Search ((afamin[Title/Abstract]) OR (Serum afamin[Title/Abstract])) OR (afamin protein[Title/Abstract])

#4) “Search #1 OR #2

#5) “Search #4 AND #3
